# Circular Whole-Transcriptome Amplification (cWTA) and mNGS Screening Enhanced by a Group Testing Algorithm (mEGA) Enable High-Throughput and Comprehensive Virus Identification

**DOI:** 10.1128/msphere.00332-22

**Published:** 2022-08-25

**Authors:** Patrick Reteng, Linh Nguyen Thuy, Mizanur Rahman, Ana Maria Bispo de Filippis, Kyoko Hayashida, Tatsuki Sugi, Gabriel Gonzalez, William W. Hall, Lan Anh Nguyen Thi, Junya Yamagishi

**Affiliations:** a Division of Collaboration and Education, International Institute for Zoonosis Control, Hokkaido University, Sapporo, Japan; b Laboratory for Molecular Diagnostics, National Institute of Epidemiology and Health, Hanoi, Vietnam; c Evercare Hospital Dhaka, Dhaka, Bangladesh; d Flavivirus Laboratory, Oswaldo Cruz Institute, Fiocruz, Rio de Janeiro, Brazil; e International Collaboration Unit, International Institute for Zoonosis Control, Hokkaido University, Sapporo, Japan; f National Virus Reference Laboratory, University College Dublin, Dublin, Ireland; g School of Medicine and Medical Research, University College Dublin, Dublin, Ireland; h Global Virus Network, Baltimore, Maryland, USA; University of Pittsburgh

**Keywords:** metagenomic, febrile illness, group testing algorithm, multiple displacement amplification, comprehensive pathogen detection

## Abstract

Metagenomic next-generation sequencing (mNGS) offers a hypothesis-free approach for pathogen detection, but its applicability in clinical diagnosis, in addition to other factors, remains limited due to complicated library construction. The present study describes a PCR-free isothermal workflow for mNGS targeting RNA, based on a multiple displacement amplification, termed circular whole-transcriptome amplification (cWTA), as the template is circularized before amplification. The cWTA approach was validated with clinical samples and nanopore sequencing. Reads homologous to dengue virus 2 and chikungunya virus were detected in clinical samples from Bangladesh and Brazil, respectively. In addition, the practicality of a high-throughput detection system that combines mNGS and a group testing algorithm termed mNGS screening enhanced by a group testing algorithm (mEGA) was established. This approach enabled significant library size reduction while permitting trackability between samples and diagnostic results. Serum samples of patients with undifferentiated febrile illnesses from Vietnam (*n* = 43) were also amplified with cWTA, divided into 11 pools, processed for library construction, and sequenced. Dengue virus 2, hepatitis B virus, and parvovirus B19 were successfully detected without prior knowledge of their existence. Collectively, cWTA with the nanopore platform opens the possibility of hypothesis-free on-site comprehensive pathogen diagnosis, while mEGA contributes to the scaling up of sample throughput.

**IMPORTANCE** Given the breadth of pathogens that cause infections, a single approach that can detect a wide range of pathogens is ideal but is impractical due to the available tests being highly specific to a certain pathogen. Recent developments in sequencing technology have introduced mNGS as an alternative that provides detection of a wide-range of pathogens by detecting the presence of their nucleic acids in the sample. However, sequencing library preparation is still a bottleneck, as it is complicated, costly, and time-consuming. In our studies, alternative approaches to optimize library construction for mNGS were developed. This included isothermal nucleic acid amplification and expansion of sample throughput with a group testing algorithm. These methods can improve the utilization of mNGS as a diagnostic tool and can serve as a high-throughput screening system aiding infectious disease surveillance.

## INTRODUCTION

Numerous infectious and noninfectious origins, in combination with nonspecific symptoms during the acute phase, make the diagnosis of febrile illnesses complicated. Hence, up to 40.6% of acute febrile illnesses in South Asia and 74.5% in Southeast Asia remain undiagnosed (1). Another study in resource-limited regions estimated this number to be between 3% and 63%, depending on the testing capacity in the regions (2). For fever of unknown origin (FUO), a diagnosis of which requires extensive workup to eliminate other possible etiologies, studies have revealed that infections account for 16% to 55% of FUO cases, suggesting that those infections were missed during the initial workup (3).

Given the large number of pathogens that may cause febrile illness, current pathogen-specific approaches, such as PCR, present a major drawback, because multiple tests are required to rule out other pathogens and this increases the possibility of missing less common pathogens. This limitation can be overcome by hypothesis-free nucleic acid detection (NAD) using metagenomic next-generation sequencing (mNGS). For mNGS, several strategies have been proposed. One of the widely employed approaches targets conserved regions known as “genetic barcodes” (i.e., 16S rRNA, 18S rRNA, and internal transcribed spacer genes) (4). In viruses, such regions exist if the suspected viruses can be narrowed down to those in a certain family or genus, severely limiting such an approach to be applied for comprehensive virus identification (5, 6). In contrast, fully comprehensive mNGS should be free from subject limitations and applicable to any pathogens. Moreover, the RNAome is ideal, given that every pathogen has RNA as a genome or transcripts. Because these RNAs are often present in only minute amounts in samples, an amplification step is required for NGS. Among the current methods, random PCR is the most straightforward (7, 8). A derived commercial product, the TransPlex whole-transcriptome amplification system (Sigma), is also available. Other commercial products, such as the direct RNA sequencing kit and PCR-cDNA sequencing kit from Oxford Nanopore Technologies, are available for single-molecule sequencing (9, 10). Collectively, differential approaches for mNGS-based diagnoses have been demonstrated; however, there are few commercial methods available to date, suggesting that improved simplicity and affordability are required for the social implementation.

Multiple displacement amplification (MDA) is an isothermal non-PCR NAD approach (5–7). It can be an alternative for a comprehensive RNAome amplification method, since it has less bias, higher reproducibility, longer amplicons, and improved simplicity. Besides, its affordability can be expected to be comparable to that for PCR. It is known that MDA is unsuitable for the amplification of RNA or small fragments (<2,000 bases), and it is biased toward circular single-stranded DNA (ssDNA) templates (11–13). This suggests that circular cDNA created by an ssDNA ligase can serve as an alternative and promising template for comprehensive RNAome amplification.

Another approach to enhance diagnosis efficiency by mNGS is sample pooling. However, usual pooling methods lose informational correspondence between input and output. In contrast, a group testing algorithm retains the informational correspondence while reducing the number of test pools to 2*n* pools for 2*^n^* samples. Indeed, the group testing algorithm has been applied to detect specific pathogens with low prevalence (14, 15). As a result, we conceived an idea of combining the group testing algorithm and the mNGS to achieve both ultra-multisample processing and comprehensive pathogen detection, simultaneously.

## RESULTS

### Amplification of total RNA by cWTA.

**FIG 1:**
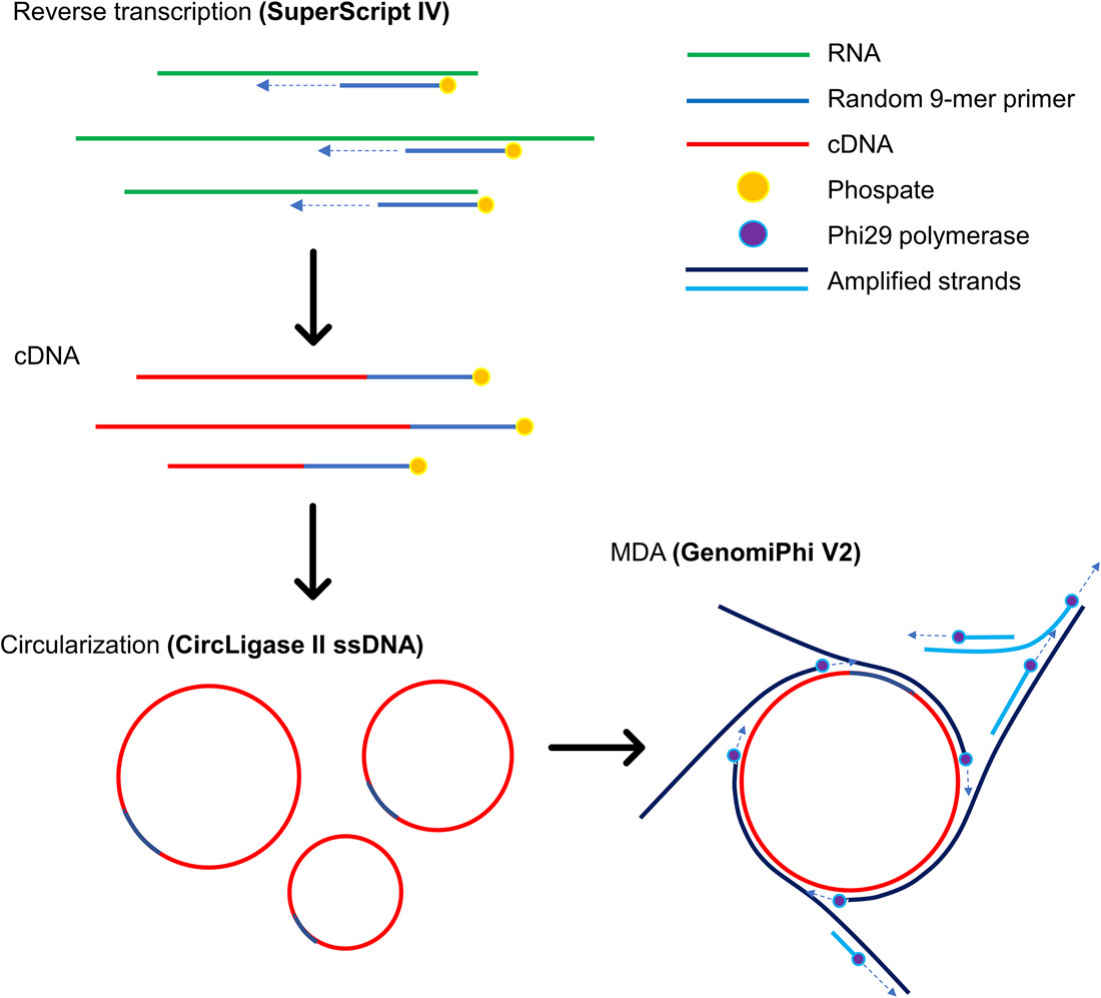
Schematic representation of the cWTA method. Random 9-mer primers with 5′-phosphate modifications were used to synthesize the cDNA and circularize it with CircLigase II ssDNA ligase with a known high affinity for ssDNA. The circularized template served as an infinite-length template for ϕ29 that will synthesize a long strand with a tandem repeat pattern. cDNA, complementary DNA; MDA, multiple displacement amplification.

**FIG 2:**
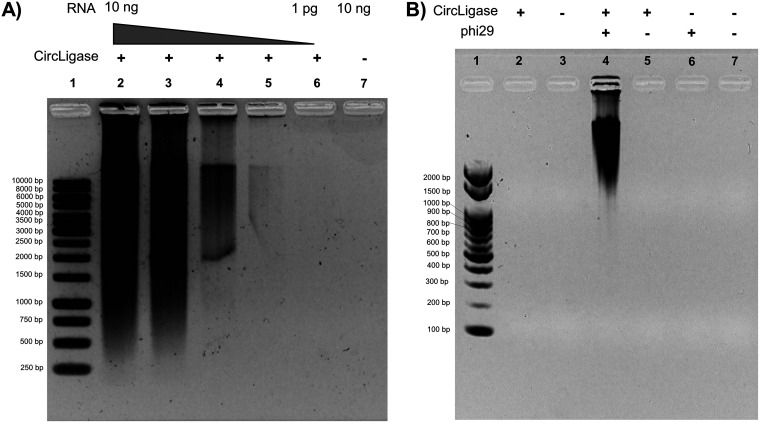
Electrophoresis results of cWTA amplicons. (A) Total RNAs purified from human foreskin fibroblast cells were used as templates. The absence of a visible band at lane 7 demonstrated that circularization is crucial for amplification. Lane 1, 1-kb marker; lanes 2 to 6, 10, 1, 0.1, 0.01, and 0.001 ng total RNA used for the reaction; lane 7, reaction containing 10 ng of total RNA without CircLigase II ssDNA ligase. (B) Circularization of the template is crucial for amplification with ϕ29. Viral RNA was extracted from spiked samples containing 10^5^ PFU/mL DENV1 and subjected to cWTA. Lane 1, 100-bp marker; lanes 2 and 3, circular (treated with ssDNA ligase) and linear template prior to amplification with ϕ29; lanes 4 and 6, samples amplified with MDA using circular (lane 4) or linear (lane 6) template; lanes 5 and 7, control samples for MDA. In the control reactions, the enzymes were simply replaced with nuclease-free water.

An initial proof-of-concept study of cWTA was successfully performed using total RNA purified from cultured cells, as shown by gel electrophoresis results (Fig. 1 and 2A). Amplicons were observed with at least 10 pg of RNA template. Meanwhile, reactions without ssDNA ligase did not show any visible bands, demonstrating that the circularization improved the amplification in MDA. A follow-up feasibility study was carried out using samples spiked with dengue virus type 1 (DENV1) virions (Fig. 2B). The experiment showed that circular ssDNA and ϕ29 are essential for amplification, as shown by the strong intensities of the bands in gel electrophoresis. The amplified products were then subjected to quantitative PCR (qPCR) and, as expected, the sample which was circularized and amplified with ϕ29 had a lower threshold cycle (*C_T_*) value (see Table S3 in the supplemental material). Hence, it can be inferred that circularizing the template with ssDNA ligase prior to MDA with ϕ29 resulted in significantly better amplification.

10.1128/msphere.00332-22.7 TABLE S3 Comparison of *C_T_* values obtained from the amplification of circularized or noncircularized template (see Fig. 1B). Download Table S3, DOCX file, 0.01 MB. Copyright © 2022 Reteng et al. 2022 Reteng et al. https://creativecommons.org/licenses/by/4.0/ This content is distributed under the terms of the Creative Commons Attribution 4.0 International license.

### Identification of viral genome sequences.

The cWTA product was then proven to be analyzable by NGS. Amplicons that originated from serum samples spiked with a series of DENV1 and DENV2 concentrations were successfully obtained (see Fig. S1A), analyzed by nanopore sequencing, and subjected to the bioinformatics pipeline. Samples containing 10^5^, 10^4^, 10^3^, and 10^2^ PFU/mL DENV1 yielded 96,502, 62,893, 91,183, and 101,591 reads after base-calling, respectively. Samples containing 10^4^, 10^3^, and 10^2^ PFU/mL DENV2 yielded 75,906, 47,000, and 80,900 reads, respectively. Reads aligned to the dengue virus genome were unambiguously detected from samples containing 10^5^ and 10^4^ PFU/mL DENV1 or 10^4^ PFU/mL DENV2 (Table 1). Reads with tandem repeat patterns presumably derived from the continuous amplification of the circular template were also observed (see Fig. S1B). In one case, the size of each tandem repeat unit was approximately 300 bp.

**TABLE 1:** Nanopore sequencing results for samples spiked with virus and clinical samples

Sample		No. of raw reads	No. (%) of reads mapped to host	No. (%) of unmapped reads	Viral reads			
					DENV1^*a*^	DENV2	CHIKV^*a*^	Other viruses
Spiked samples	DENV1 10^5^	96,502	45,309 (46.95)	51,193 (53.05)	35	0	0	0
	DENV1 10^4^	62,893	46,859 (74.51)	16,034 (25.49)	8	0	0	0
	DENV1 10^3^	91,183	68,702 (75.35)	22,481 (24.65)	1	0	0	0
	DENV1 10^2^	101,591	78,776 (77.54)	22,815 (22.46)	0	0	0	0
	DENV2 10^4^	75,906	34,125 (44.96)	41,781 (55.04)	0	36	0	0
	DENV2 10^3^	47,000	36,622 (77.92)	10,378 (22.08)	0	1	0	0
	DENV2 10^2^	80,900	63,060 (77.95)	17,840 (22.05)	0	0	0	0
Clinical samples	Bangladesh 01	18,539	16,596 (89.52)	1,943 (10.48)	0	3	0	0
	Bangladesh 02	6,789	6,048 (89.09)	741 (10.91)	0	3	0	0
	Fiocruz 01	11,068	8,854 (80.00)	2,214 (20.00)	0	0	1,576	0
	Fiocruz 02	9,868	8,930 (90.49)	938 (9.51)	0	0	417	0
	Fiocruz 03	10,148	9,372 (92.35)	776 (7.65)	0	0	1	0
	Fiocruz 04	11,897	10,983 (92.32)	914 (7.68)	0	0	4	0
	Fiocruz 05	7,266	6,955 (95.72)	311 (4.28)	0	0	15	0
	Fiocruz 06	11,190	10,715 (95.76)	475 (4.24)	0	0	43	0

a DENV, dengue virus; CHIKV, chikungunya virus.

10.1128/msphere.00332-22.1 FIG S1 Results of cWTA with samples containing viral particles. (A) cWTA successfully amplified RNA in the artificial samples. Amplification was observed in FBS spiked with DENV1 and DENV2 virions. Lane 1, 100-bp marker; lanes 2, 3 and 4, 10^5^, 10^4^, and 10^3^ PFU/mL DENV1; lanes 5, 6, and 7, 10^4^, 10^3^, and 10^2^ PFU/mL DENV2. (B) Visualization of the alignment result of one read obtained from Nanopore sequencing, showing that the 6-kbp Nanopore read consists of an approximately 300-bp tandem repeat. The trapezoids represent projection of the subject onto the query. The color represents the quality of alignment, with darker shades indicating high scoring hits (based on e-value and bit score). The BLAST result was visualized with Kablammo (37). Download FIG S1, TIF file, 2.8 MB. Copyright © 2022 Reteng et al. 2022 Reteng et al. https://creativecommons.org/licenses/by/4.0/ This content is distributed under the terms of the Creative Commons Attribution 4.0 International license.

Circular whole-transcriptome amplification was then performed in combination with nanopore sequencing in two sets of clinical samples. First, two samples that tested positive for DENV in Bangladesh were subjected to cWTA and nanopore sequencing. Base-calling yielded 18,539 and 6,789 reads, and 3 reads from each sample were homologous to DENV2 (Table 1). The second set of samples originated from Brazil. Six serum samples were subjected to the workflow as described above for nanopore sequencing. Base-calling and debarcoding yielded raw reads ranging from 7,266 to 77,877. Reads homologous to chikungunya virus (CHIKV) were obtained from six samples. When mapped to the CHIKV reference genome, two samples yielded more than 90% coverage (see Fig. S2). This finding clearly demonstrated that cWTA combined with NGS is clearly applicable for the detection of viral pathogens in clinical samples.

10.1128/msphere.00332-22.2 FIG S2 Mapping of viral reads obtained from Illumina or Nanopore sequencing to the respective viral reference genome. The accession numbers for the reference genomes were NC_001477 (DENV1), NC_001474 (DENV2), NC_004162 (CHIKV), NC_000883 (B19), NC_003977 (HBV), and NC_001802 HIV-1. DENV, dengue virus; CHIKV, chikungunya virus; B19, parvovirus B19; HBV, hepatitis B virus; HIV, human immunodeficiency virus. Download FIG S2, TIF file, 2.8 MB. Copyright © 2022 Reteng et al. 2022 Reteng et al. https://creativecommons.org/licenses/by/4.0/ This content is distributed under the terms of the Creative Commons Attribution 4.0 International license.

### Parallel viral identification by application of the group testing algorithm.

The cWTA, in combination with mEGA, was then applied to detect viral pathogens in serum samples obtained from Vietnam. A total of 43 serum samples from patients with acute fever and one sample with a positive control serum (sample 67), which was confirmed to be DENV1 by a commercial reverse transcription-qPCR (RT-qPCR) assay, were subjected to cWTA. Illumina MiSeq was applied to this sample set to acquire more reads and confirm that the amplicon was also readable by the Illumina sequencing platform. With mEGA, instead of constructing 44 individual libraries, the number of libraries was compressed to a total of 11 libraries (see Table S1). These libraries were divided into five pool groups (represented by columns in Table S1), and each pool group consisted of two or three pools. Total raw reads obtained ranged from 2,852,907 to 5,054,500 (median, 4,260,704) (Table 2). On average, for 25.61% ± 1.02% (mean ± standard deviation) of raw reads the BLAST search step was completed, whereas most reads were dropped during the host decontamination process (57.97% ± 0.99%) and the quality control step (16.41% ± 0.5%). Among the 11 libraries, 1a, 2a, 3a, 4b, and 5b contained DENV1 reads (see Fig. S3A). The only sample pooled into these libraries was sample 67, suggesting that the sample was DENV1 positive. As mentioned above, sample 67 was added to the system as a positive control and the result was in accordance with our expectations. Reads homologous to DENV2 were detected in pools 1a, 2a, 3a, 4a, and 5b (see Fig. S3B), which indicated that these DENV2 reads originated from sample 8. In addition to these two samples, reads homologous to human parvovirus B19 and hepatitis B virus (HBV) were detected in samples 98 and 99, respectively (see Fig. S3C and D). Interestingly, reads homologous to parvovirus B19 were sufficient to cover the entire genome (see Fig. S2). It was assumed that this observation was due to the high viral load of parvovirus B19 in the serum sample, consistent with previous reports (16). Finally, reads homologous to HIV-1 were detected in samples 96 and 97 (see Fig. S3E).

**TABLE 2:** Total raw reads obtained from sequencing, reads dropped during downstream analysis, viral reads, and confirmation PCR results for Vietnam samples using cWTA and mEGA^*a*^

Pool	Raw reads	No. (%) of reads dropped from QC	No. (%) of reads mapped to host	No. (%) of unmapped reads	Viral reads							
					DENV1	DENV2	B19	HIV1	HBV	TTV8	TTV24	V9
1a	3,548,149	594,485 (16.75)	2,025,030 (57.07)	928,634 (26.17)	9	84	4	0	0	0	0	0
1b	4,776,701	780,689 (16.34)	2,815,856 (58.95)	1,180,156 (24.71)	0	0	47,820	2	3	0	1	0
2a	2,852,907	471,288 (16.52)	1,632,297 (57.22)	749,322 (26.27)	5	101	34,861	8	5	0	1	0
2b	3,960,684	639,447 (16.14)	2,348,298 (59.29)	972,939 (24.56)	0	0	0	0	0	1	0	0
3a	3,616,010	600,703 (16.61)	2,055,922 (56.86)	959,385 (26.53)	10	37	5	0	0	0	0	0
3b	5,054,500	859,593 (17.01)	2,919,607 (57.76)	1,275,300 (25.23)	0	0	73,521	12	3	0	0	1
4a	4,861,811	839,593 (17.27)	2,785,501 (57.29)	1,236,717 (25.44)	0	137	31	4	0	0	0	0
4b	4,664,334	737,011 (15.80)	2,731,045 (58.55)	1,196,278 (25.65)	22	0	46,792	0	1	0	0	0
5a	4,782,575	768,445 (16.07)	2,682,222 (56.08)	1,331,908 (27.85)	0	0	96,198	0	0	0	0	1
5b	3,964,608	618,340 (15.60)	2,367,500 (59.72)	978,768 (24.69)	9	134	0	1	2	0	0	0
5c	4,260,704	697,628 (16.37)	2,510,115 (58.91)	1,052,961 (24.71)	0	0	0	9	0	0	0	0
Sample ID					67	8	98	96/97	99	UD	UD	UD
Confirmation by PCR					+	+	+	−	+	NT	NT	NT

a The Sample ID refers to the source of viral reads, which was traced based on the pools containing viral sequence (see Table S1, Figure S3). UD, undetermined; NT, not tested.

10.1128/msphere.00332-22.3 FIG S3 Sequencing results from each pool in mEGA (see Table S1 for the pooling strategy). (A) Result for sample 67, the positive control. The matrix shows the number DENV1 reads obtained from sequencing. (B) DENV2 reads obtained from sequencing. (C) B19 reads obtained from sequencing. (D) HBV reads obtained from sequencing. (E) HIV-1 reads obtained from sequencing. Numbers highlighted in red show positive pools. Rows outlined in red show where all five pools have viral reads. Samples were confirmed by pathogen-specific PCR. DENV, dengue virus; B19, parvovirus B19; HBV, hepatitis B virus; HIV, human immunodeficiency virus; NTC, no-template control. Download FIG S3, TIF file, 2.1 MB. Copyright © 2022 Reteng et al. 2022 Reteng et al. https://creativecommons.org/licenses/by/4.0/ This content is distributed under the terms of the Creative Commons Attribution 4.0 International license.

10.1128/msphere.00332-22.5 TABLE S1 Pooling strategy used for samples collected in Vietnam. Download Table S1, DOCX file, 0.02 MB. Copyright © 2022 Reteng et al. 2022 Reteng et al. https://creativecommons.org/licenses/by/4.0/ This content is distributed under the terms of the Creative Commons Attribution 4.0 International license.

Samples positive for DENV1, DENV2, HBV, parvovirus B19, and HIV-1 were subjected to pathogen-specific PCR and Sanger sequencing for validation; amplicons with more than 95% sequence similarity to each target were obtained, except for HIV-1 (see Fig. S3). Further validation for HIV-1 was conducted by designing a nested PCR system targeting the region covered by mapped sequence reads (see Table S2 and Fig. S2). Amplicons were obtained from sample 97 but not sample 96 (see Fig. S3E), both of which were subsequently sequenced. When subjected to a BLAST search, the amplicon sequence was similar to that of an HIV-1 isolate, with 92.49% identity. This suggests that the sequence could have originated from an undescribed retrotransposon or a human endogenous retrovirus, but it cannot be excluded that these sequences were from a novel lentivirus, including an unidentified strain of HIV-1, considering the genetic diversity of the HIV-1 Gag gene (up to 35%) (17).

10.1128/msphere.00332-22.6 TABLE S2 List of primers used for pathogen-specific PCR. Download Table S2, DOCX file, 0.01 MB. Copyright © 2022 Reteng et al. 2022 Reteng et al. https://creativecommons.org/licenses/by/4.0/ This content is distributed under the terms of the Creative Commons Attribution 4.0 International license.

In addition, reads homologous to erythrovirus V9 and torque teno virus (TTV) were detected. Erythrovirus V9 is closely related to parvovirus B19; in the BLAST search results, the erythrovirus V9 reads aligned to a region that shared more than 99% similarity to parvovirus B19. There were also reads for which the alignment search resulted in hits for both parvovirus B19 and erythrovirus V9, with an identical bit score and e-value, particularly in regions where the two viruses share similarity. As it was impossible to tell them apart, these reads (1,198 reads, 0.4% of all parvovirus B9 and erythrovirus V9 reads) were filtered out. As for the reads homologous to TTV, these reads could only be detected in a limited number of pools; thus, the sample origin of the reads could not be determined.

It is also known that index hopping is a common issue when performing multiplex sequencing. We applied 0.1% as the index hopping rate and Poisson process to estimate the possible number of reads caused by the index hopping (18, 19). Then, the number of reads (μ) that resulted in an upper cumulative Poisson probability *P*(*x* ≥ μ) of <0.01 was determined as the cutoff value. Based on this approach, the cutoff value for B19 was 341 reads. As such, reads in pools 1a, 3a, and 4a were possible migrations from the other pools (see Table S4).

10.1128/msphere.00332-22.8 TABLE S4 Sequencing reads from the pooled experiment aligned to parvovirus B19 and the subsequent threshold calculation. Download Table S4, DOCX file, 0.01 MB. Copyright © 2022 Reteng et al. 2022 Reteng et al. https://creativecommons.org/licenses/by/4.0/ This content is distributed under the terms of the Creative Commons Attribution 4.0 International license.

## DISCUSSION

In the present study, a novel comprehensive RNAome amplification method, cWTA, was developed by integrating MDA with single-stranded cDNA circularization. It is known that MDA is less efficient for short templates because there are fewer priming sites, which leads to fewer hyperbranching cycles (11). However, circularization of the short template has been shown to successfully amplify short nucleotide sequences, such as small RNAs (20). Nevertheless, this method requires a sequence-specific splint oligonucleotide to allow circularization with T4 DNA ligase; thus, the use of this technique for comprehensive amplification is limited. In contrast, our method directly ligates ssDNA synthesized from RT by using a unique DNA ligase with enough efficiency for the ssDNA substrate. Through a series of validations, we demonstrated that the circularization was critical for improving MDA. Given that the genome or transcripts of pathogens are often limited in specimens, such as serum or plasma, a nonspecific amplification step is crucial for pathogen detection. Indeed, the workflow was able to detect reads homologous to DENV2 and CHIKV from clinical samples from Bangladesh and Brazil, supporting the feasibility and reliability of the system for clinical samples.

Given that the nanopore sequencer is portable, stand-alone, and has low implementation costs, the combination of cWTA and the nanopore sequencer in field settings or local clinics with limited resources is promising. To increase the scalability, cWTA can be combined with a high-throughput, high-accuracy Illumina sequencing platform. However, labor-, time-, and cost-consuming library construction will raise another bottleneck for large-scale application. To address this potential obstacle, a systematic pooling algorithm known as group testing was implemented. With mEGA, 2*^n^* samples will be pooled into 2*n* sequencing libraries, while keeping trackability among samples and results. A proof-of-concept study of this method was performed with clinical samples from Vietnam, and viruses with different genomic characteristics (single-stranded RNA, ssDNA, and partially double-stranded DNA) were successfully detected without prior knowledge.

Several limitations related to the cWTA approach were noted. First, because cWTA does not specifically target the pathogens’ genomes, more than half of the reads (57.92% ± 1.17%) were aligned to the host genome. This is a common issue in mNGS and can be improved by depleting abundant host RNA in the sample. Second, one inconclusive case (HIV-1) was observed. This downside can be overcome by future improvements of the data set for human and pathogen genomes. Finally, there is insufficient evidence demonstrating the comprehensiveness of the method. Thus, sensitivity and preference for each virus remains to be validated. To date, there are comprehensive amplification methods for the detection of the RNAome, such as random PCR amplification or sequence-independent single-primer amplification (8), multiple annealing and looping-based amplification (21), and linker amplification shotgun libraries (22). These are comparable to cWTA, but their sensitivities for each virus have not been demonstrated. This is a critical point if these sequencing-based diagnoses are to be widely implemented; thus, they should be individually validated in future studies.

The group testing algorithm was originally developed for the effective diagnosis syphilitic antigen in 1974 (14). Currently, a derivative of the algorithm called hypercube algorithm was also applied for the screening of SARS-CoV-2, for which 3^n^ samples were pooled into 3n pools (15). In essence, the pooling algorithm is similar to that used in our study, where the number of libraries grew in a logarithmic fashion (see Fig. S4 in the supplemental material). However, our approach expands the number of possible pathogens that can be identified, even without prior knowledge. Thus, it is possible to optimize mEGA for large-scale screening, such as identification of potential pathogens from FUO patients in hospital settings, retrospective screening of biobank samples for prezoonotic pathogens, and routine testing for blood transfusions. One concern is that the sample tracing might be limited when there are three or more samples containing the same pathogen in a batch. Nevertheless, this disadvantage is not critical, because the major targets of the method are rare, undiagnosed, neglected pathogens. In addition, if enough reads were obtained for pathogens, genomic polymorphism could be employed to discriminate the same pathogens that originated from different samples.

10.1128/msphere.00332-22.4 FIG S4 Number of libraries needed to be constructed in the systematic pooling experiments. Download FIG S4, TIF file, 1.2 MB. Copyright © 2022 Reteng et al. 2022 Reteng et al. https://creativecommons.org/licenses/by/4.0/ This content is distributed under the terms of the Creative Commons Attribution 4.0 International license.

In this study, we demonstrated that cWTA was applicable for amplification of the RNAome. This study also validated mEGA, an application of the group testing algorithm for large-scale comprehensive pathogen detection, which reduced the number of sequencing libraries that needed to be constructed. Given the affordability and portability of the nanopore sequencer and the isothermal nature of cWTA, the workflow is feasible for more peripheral laboratories with limited settings. In contrast, mEGA using the Illumina platform will improve the scalability suitable for large-scale screening. Finally, although this paper is focused only on viruses, the workflow can theoretically provide broad-range detection of pathogens, including parasites and bacteria. Taken together, this approach and workflow may provide one of the better practices in NAD for a comprehensive approach for pathogen identification.

## MATERIALS AND METHODS

### Circular whole-transcriptome amplification.

The feasibility of the system was evaluated using total RNA. The RNA was purified from cultured human foreskin fibroblast cells with TRIzol (Thermo Fisher Scientific) and Direct-zol RNA kits (Zymo Research). DNase treatment was conducted according to the manufacturer’s instructions. Total RNA was subjected to RT using Superscript IV (Invitrogen). Random 9-mer primers with a phosphate modification at the 5′-end (N9P primer) were used for the RT reaction. RT reactions were carried out in a total volume of 20 μL using 1 pg to 10 ng of template RNA according to the manufacturer’s instructions, except for the N9P primer (for which a final concentration of 1.25 μM was used instead of 2.5 μM). Incubations were carried out in a thermal cycler. Equal amounts of AmPure XP beads were added to the cDNA, purified according to the manufacturer’s instructions, and eluted in 15 μL of nuclease-free water.

The ligation reactions were carried out with CircLigase II ssDNA ligase (Lucigen), in a total volume of 20 μL consisting of 12.5 μL purified cDNA, 2 μL reaction buffer, 1 μL MnCl_2_, 0.5 μL CircLigase II ssDNA ligase, and nuclease-free water. The mixture was then incubated at 60°C for 1 h and at 80°C for 10 min.

Multiple displacement amplification was performed with the GenomiPhi V2 (Cytiva). Specifically, 1 μL of ligated sample was mixed with 9 μL sample buffer. The sample buffer contained random hexamer primer. To prime the random hexamers, the mixture was incubated at 95°C for 5 min and immediately placed on ice. A 9-μL aliquot of reaction buffer and 1 μL ϕ29 enzyme were then added to the mixture and then incubated at 30°C for 90 min and 65°C for 5 min. The products (1-μL volume) were then visualized on 1.5% agarose gels.

The feasibility studies were then continued with artificial samples. Fetal bovine serum (FBS) was spiked with viral particles of DENV1 at final concentrations of 10^3^, 10^4^, and 10^5^ PFU/mL and DENV2 at final concentrations of 10^2^, 10^3^, and 10^4^ PFU/mL. RNA was extracted from the artificial samples using a QIAamp viral RNA minikit (Qiagen) according to the manufacturer’s instructions. The input volume was 140 μL, and the final elution volume was 60 μL. The extracted RNA was then processed for RT, ligation, and MDA according to the above protocols. The products were subjected to sequencing with the MinION Flowcell (see below).

To confirm that the circular template increased amplification efficiency, RNA extracted from the spiked FBS sample (10^5^ PFU/mL) was subjected to cWTA. In the untreated samples, CircLigase II ssDNA ligase or ϕ29 was replaced with nuclease-free water. qPCR was carried out using a previously described primer set for flaviviruses (23). The reaction was carried out using the Kapa *Taq* Extra PCR kit (Kapa Biosystems) in a total volume of 15 μL containing 1.5 μL template, 250 nM each primer (DEN4F and Flavivirus S_1, 500 nM primer flavivirus AS2), and 0.75 μL EvaGreen (Biotium). The qPCR was performed in a Bio-Rad CFX96 apparatus with temperature conditions for the PCR as follows: 94°C for 30 s, followed by 43 cycles of 94°C, 53°C, and 72°C for 30 s each, and final extension at 72°C for 5 min, followed by a melting curve analysis.

### Clinical samples.

Human serum samples were obtained from Bangladesh, Brazil, and Vietnam. Two samples from Bangladesh and six samples from Brazil were subjected to cWTA as described above. Extraction of RNA was performed using a QIAmp viral RNA minikit (Qiagen). The amplicons were sequenced with nanopore sequencing (with barcodes), as described in the next section. Serum samples from Bangladesh were known to be DENV2 positive, as confirmed by RT-qPCR at the Evercare Hospital (Dhaka, Bangladesh). Serum samples from Brazil were collected from febrile patients from 2017 to 2018 and stored at the Flavivirus Reference Laboratory, Oswaldo Cruz Institute (Rio de Janeiro, Brazil). For validation of mNGS screening enhanced by the group testing algorithm (mEGA), 44 serum samples collected from patients in Nam Dinh Province, Vietnam, from May 2017 to May 2019, were included. These serum samples were stored at −80°C prior to RNA extraction. Extraction of RNA was carried out using a QIAamp viral RNA minikit (Qiagen). Samples were subjected to cWTA and later to Illumina sequencing. One sample positive for DENV1 confirmed by RT-qPCR (CDC DENV-1-4 Real-Time RT-PCR multiplex assay) was included as a positive control. Serum samples were collected and stored by the National Institute of Hygiene and Epidemiology (NIHE), Hanoi, Vietnam. Ethical approvals were obtained from Apollo Hospital (now Evercare Hospital) in Dhaka (ERC 16/2018-2), Fiocruz (90249218.6.1001.5248: 2.998.362), NIHE (351/QD-VSDTTU), and Hokkaido University (Jinjyu1-3, Jinjyu30-4, and Jinjyu30-1).

### Library preparation for nanopore sequencing.

Samples from Bangladesh and Brazil were prepared using the library kit SQK-LSK108 (Oxford Nanopore Technologies) according to the manufacturer’s instructions. For samples spiked with viral particles, the libraries were prepared using library kits SQK-RLB001 and SQK-LSK109. When necessary, the libraries were barcoded, using the barcoding kit NBD-LSK103 (Oxford Nanopore Technologies). Sequencing was carried out on a MinION Flowcell (version 9.4), per the manufacturer’s instructions.

### Library preparation for Illumina MiSeq paired-end sequencing.

Prior to pooling, 500 ng of cWTA product from each sample was diluted with nuclease-free water to a final volume of 10 μL. Eleven sample pools were constructed according to the systematic diagram (see Table S1 in the supplemental material) consisting of 1.5 μL from each diluted sample. From each pool, 200 ng of DNA was used to construct the sequencing library. Sequencing libraries were prepared with TruSeq Nano DNA Library prep (Illumina) according to the manufacturer’s instructions. Libraries were then quantified with a fluorometer, and the quality was assessed with Bioanalyzer (Agilent). The concentrations of the libraries were adjusted to a final concentration of 4 nM. Sequencing was performed using the Illumina MiSeq to generate 2 × 150-bp, paired-end reads.

### Bioinformatic pipeline.

Raw data obtained from nanopore sequencing were base-called with Albacore version 1.1.2 or Guppy version 3.2.4 (Oxford Nanopore Technologies). The *q* score threshold was set to 60. Debarcoding was carried out using Guppy or Albacore, with default parameters. Reads were then mapped against the host sequence (Homo sapiens [GCF_000001405.39] or Bos taurus [GCF_002263795.1]) using Minimap2 (24) version 2.17. Unmapped reads were then extracted using SAMtools (25) version 1.10.

For Illumina reads, adapter sequences were removed, and quality filtering of bases was carried out with Cutadapt (26) version 2.10. Reads passing filters were then processed with Trimmomatic (27) version 0.3.9 for second adapter removal and quality filtering. The parameters set for Trimmomatic were as follows: leading 3, trailing 3, sliding window [4, 15], and minimal length 36. Surviving sequences were then filtered for low-complexity sequencing with Komplexity (28) version 0.3.6 (the complexity score threshold set to 0.55). Filtered reads were then mapped against the human sequence (GCF_000001405.39) using Bowtie2 (29) version 2.2.4. Unmapped reads were extracted using SAMtools (25) with the following flags: -f 12 -F 256.

Only reads decontaminated from human reads were subjected to the first alignment search using BLAST (30) version 2.9.0 against the viral databases as described previously (31), with the word size parameter set to 11. In this database, sequence regions in the viral genome that highly resemble human or bacterial genomes are masked and redundant viral genome (≥90% identical) is concatenated. Based on the results of the first BLAST search, sequences aligned to the database with an e-value <1.0e−10 were extracted and subjected to the second BLAST search. The database for the second BLAST search was comprised of the viral sequence database as above, human sequences, and representative prokaryote genomes (31). The sequences were assigned to the top-hit feature based on the bit score. If the bit score happened to be a tie, then the read was discarded. If reads from a paired-end sequence were assigned to different viruses, the reads would be exempted from the final count. In the case of nanopore sequencing reads, hits with the highest bit score were counted.

### Cross-validation.

To validate the sequencing results, samples with certain virus reads were subjected to pathogen-specific PCR. The list of primers used for cross-validation can be found in Table S2 in the supplemental material (32–36). Each 10-μL reaction mixture consisted of 1 μL template, 2 μL *Taq* buffer, 0.8 μL deoxynucleotide trisphosphates (5 mM each), 0.05 μL *Taq* Hot Start polymerase (TaKaRa), and 200 to 500 nM each primer (forward and reverse). cDNA was used as the template, except for HIV-1 positive samples, due to the limited amount of the remaining cDNA. The cycling program can be found in Table S2. For seminested or nested PCR, the template for the second PCR was 100 times the diluted first PCR product. As much as 1 μL of PCR product was visualized with gel electrophoresis on 1.5% agarose gels. Furthermore, all PCR amplicons were cloned into the pGEM-T vector (Promega) and transformed into Escherichia coli D5Hα. The amplicons were cloned to the plasmids due to some amplicons being too short to be sequenced using Sanger sequencing. The cloned plasmids were then subjected to PCR with the Sp6 and T7 primers. The amplicons were then processed for Sanger sequencing.
