# Supplementary material for: Circular Whole-Transcriptome Amplification (cWTA) and mNGS Screening Enhanced by a Group Testing Algorithm (mEGA) Enable High-Throughput and Comprehensive Virus Identification
Source: mSphere. 2022 Aug 25;7(5):e00332-22. doi: 10.1128/msphere.00332-22 (PMC9599668; doi:10.1128/msphere.00332-22)
Supplement: TABLE S3 [file msphere.00332-22-s0007.docx]

**Table S3.** Comparison of CT obtained from the amplification of circularized or noncircularized template (see Fig. 1B).

| CircLigase | Phi29 | CT value | | Mean |
| --- | --- | --- | --- | --- |
|  |  | rep1 | rep2 |  |
| + | + | 32.22 | 31.8 | 32.01 |
| + | - | ND | ND | ND |
| - | + | 37.01 | 36.95 | 36.98 |
| - | - | ND | ND | ND |

ND, not detected; CT, cycle threshold; rep, technical replicate.
