# Supplementary figures and images for: Circular Whole-Transcriptome Amplification (cWTA) and mNGS Screening Enhanced by a Group Testing Algorithm (mEGA) Enable High-Throughput and Comprehensive Virus Identification
Source: mSphere. 2022 Aug 25;7(5):e00332-22. doi: 10.1128/msphere.00332-22 (PMC9599668; doi:10.1128/msphere.00332-22)

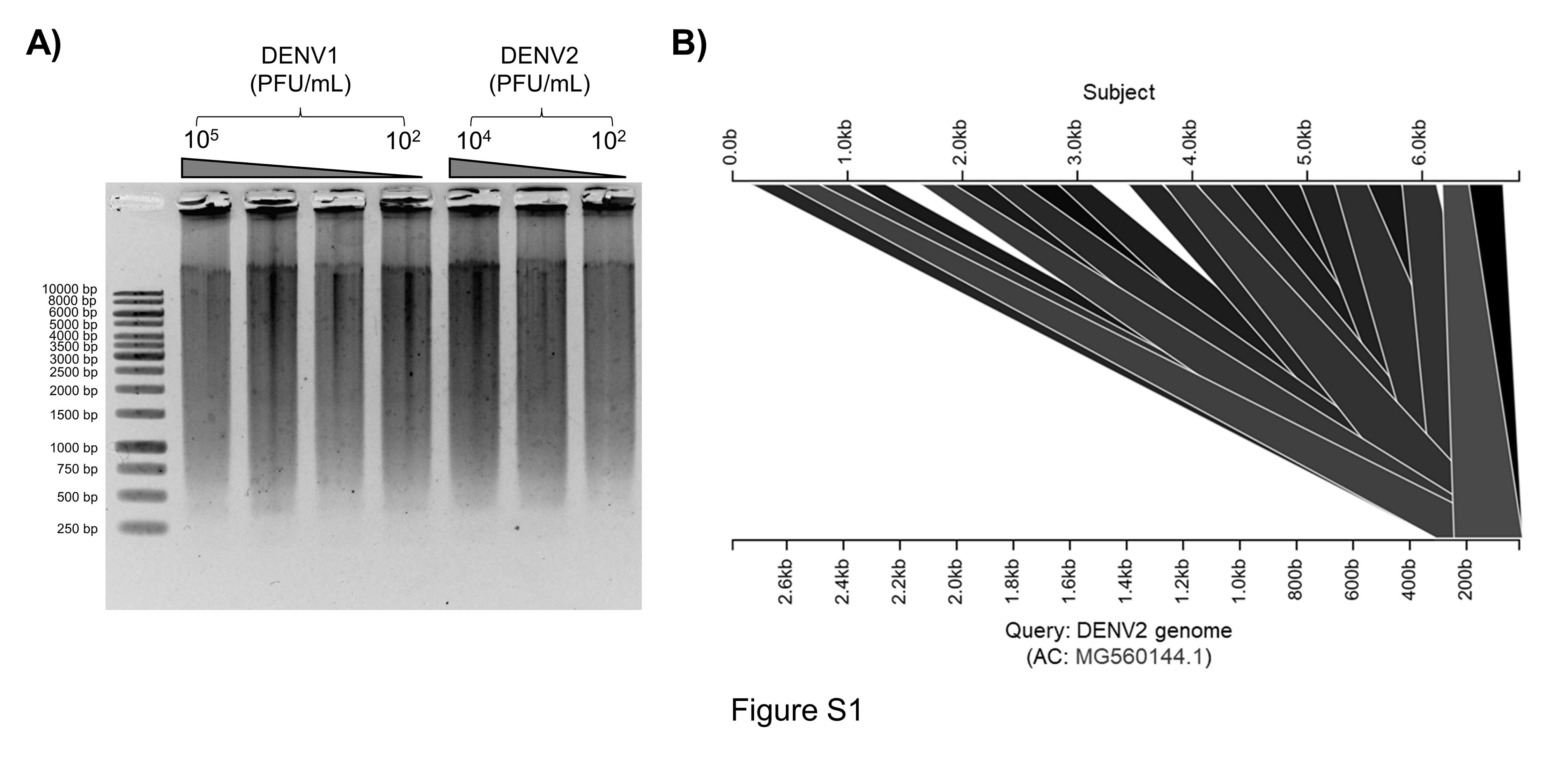

Supplement: FIG S1 [file msphere.00332-22-s0001.tif]

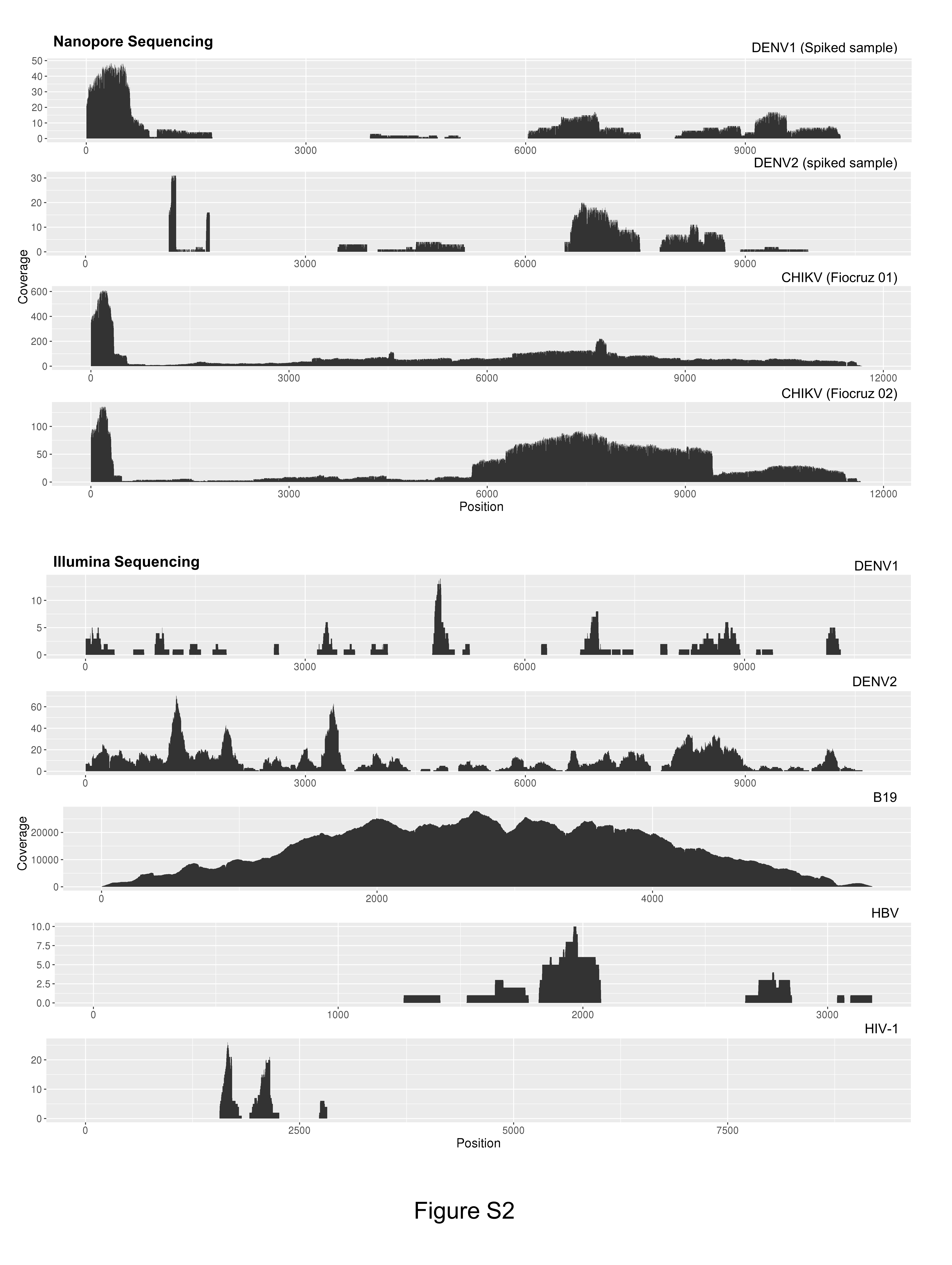

Supplement: FIG S2 [file msphere.00332-22-s0002.tif]

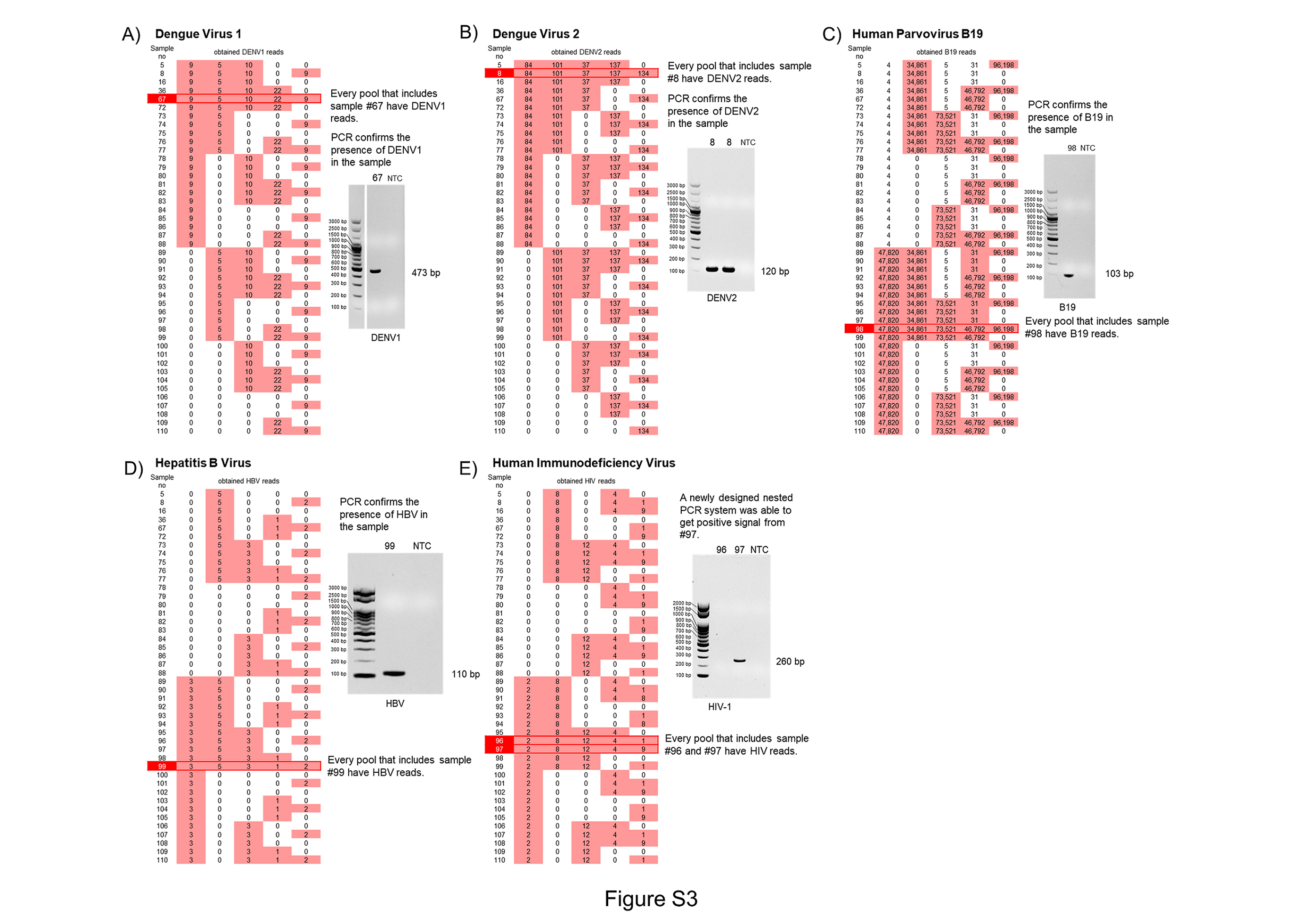

Supplement: FIG S3 [file msphere.00332-22-s0003.tif]

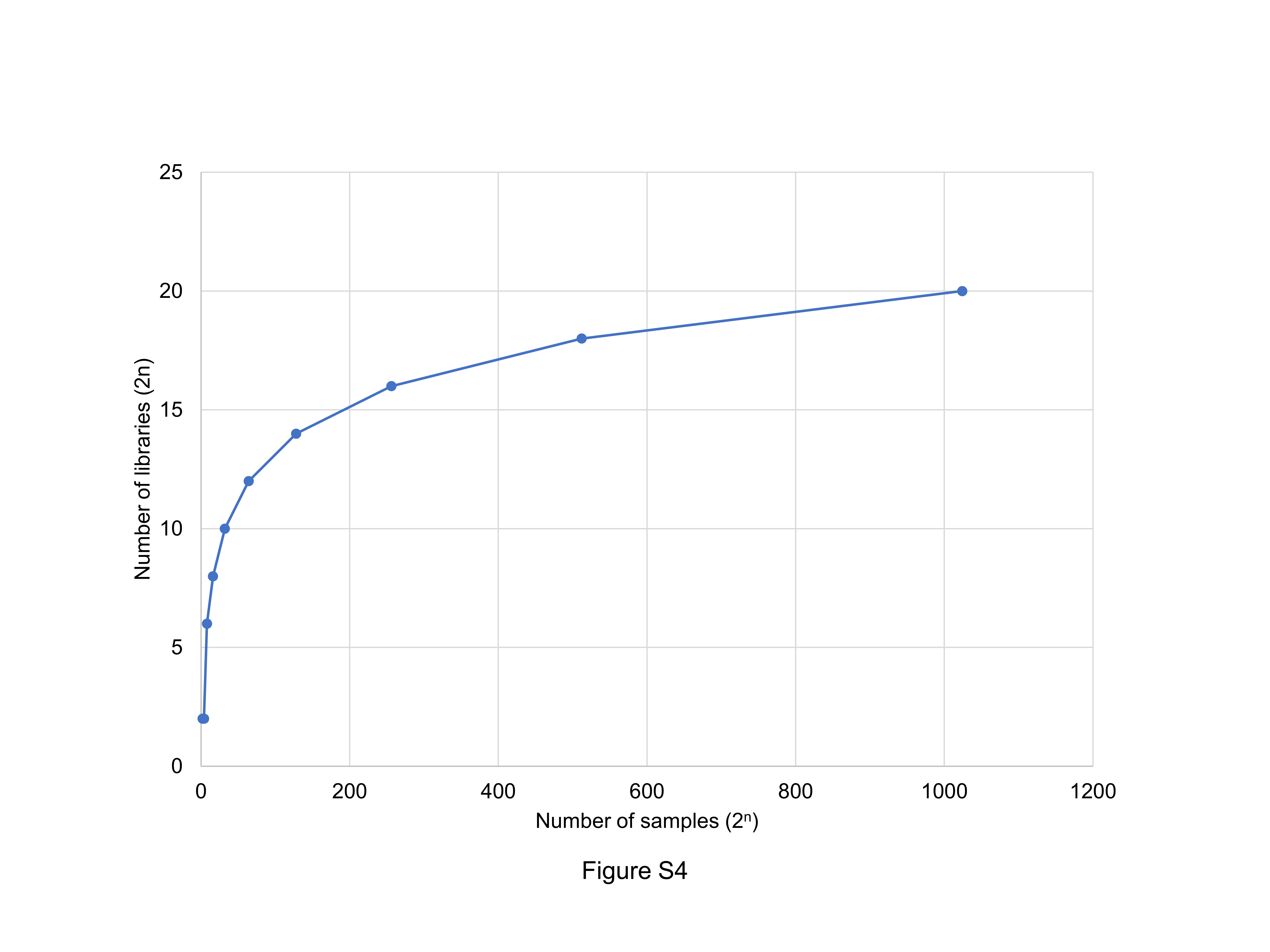

Supplement: FIG S4 [file msphere.00332-22-s0004.tif]
