# Supplementary material for: Circular Whole-Transcriptome Amplification (cWTA) and mNGS Screening Enhanced by a Group Testing Algorithm (mEGA) Enable High-Throughput and Comprehensive Virus Identification
Source: mSphere. 2022 Aug 25;7(5):e00332-22. doi: 10.1128/msphere.00332-22 (PMC9599668; doi:10.1128/msphere.00332-22)
Supplement: TABLE S1 [file msphere.00332-22-s0005.docx]

**Table S1.** Pooling strategy used for samples collected in Vietnam^a^

| Sample no | Pool pattern | | | | |  | Sample no | Pool pattern | | | | |
| --- | --- | --- | --- | --- | --- | --- | --- | --- | --- | --- | --- | --- |
| 5 | 1a | 2a | 3a | 4a | 5a |  | 89 | 1b | 2a | 3a | 4a | 5a |
| 8 | 1a | 2a | 3a | 4a | 5b |  | 90 | 1b | 2a | 3a | 4a | 5b |
| 16 | 1a | 2a | 3a | 4a | 5c |  | 91 | 1b | 2a | 3a | 4a | 5c |
| 36 | 1a | 2a | 3a | 4b | 5a |  | 92 | 1b | 2a | 3a | 4b | 5a |
| 67 | 1a | 2a | 3a | 4b | 5b |  | 93 | 1b | 2a | 3a | 4b | 5b |
| 72 | 1a | 2a | 3a | 4b | 5c |  | 94 | 1b | 2a | 3a | 4b | 5c |
| 73 | 1a | 2a | 3b | 4a | 5a |  | 95 | 1b | 2a | 3b | 4a | 5a |
| 74 | 1a | 2a | 3b | 4a | 5b |  | 96 | 1b | 2a | 3b | 4a | 5b |
| 75 | 1a | 2a | 3b | 4a | 5c |  | 97 | 1b | 2a | 3b | 4a | 5c |
| 76 | 1a | 2a | 3b | 4b | 5a |  | 98 | 1b | 2a | 3b | 4b | 5a |
| 77 | 1a | 2a | 3b | 4b | 5b |  | 99 | 1b | 2a | 3b | 4b | 5b |
| 78 | 1a | 2b | 3a | 4a | 5a |  | 100 | 1b | 2b | 3a | 4a | 5a |
| 79 | 1a | 2b | 3a | 4a | 5b |  | 101 | 1b | 2b | 3a | 4a | 5b |
| 80 | 1a | 2b | 3a | 4a | 5c |  | 102 | 1b | 2b | 3a | 4a | 5c |
| 81 | 1a | 2b | 3a | 4b | 5a |  | 103 | 1b | 2b | 3a | 4b | 5a |
| 82 | 1a | 2b | 3a | 4b | 5b |  | 104 | 1b | 2b | 3a | 4b | 5b |
| 83 | 1a | 2b | 3a | 4b | 5c |  | 105 | 1b | 2b | 3a | 4b | 5c |
| 84 | 1a | 2b | 3b | 4a | 5a |  | 106 | 1b | 2b | 3b | 4a | 5a |
| 85 | 1a | 2b | 3b | 4a | 5b |  | 107 | 1b | 2b | 3b | 4a | 5b |
| 86 | 1a | 2b | 3b | 4a | 5c |  | 108 | 1b | 2b | 3b | 4a | 5c |
| 87 | 1a | 2b | 3b | 4b | 5a |  | 109 | 1b | 2b | 3b | 4b | 5a |
| 88 | 1a | 2b | 3b | 4b | 5b |  | 110 | 1b | 2b | 3b | 4b | 5b |

^a^The number represents the pool groups each group consist of two or three different pools (labelled with a, b, or c). Every sample was added to five different pools, as represented by the pool name in each column. For example, pool 1a consists of sample number 5 to 88 and sample number 5 was added to pool 1a, 2a, 3a, 4a, and 5a.
