# Supplementary material for: Circular Whole-Transcriptome Amplification (cWTA) and mNGS Screening Enhanced by a Group Testing Algorithm (mEGA) Enable High-Throughput and Comprehensive Virus Identification
Source: mSphere. 2022 Aug 25;7(5):e00332-22. doi: 10.1128/msphere.00332-22 (PMC9599668; doi:10.1128/msphere.00332-22)
Supplement: TABLE S2 [file msphere.00332-22-s0006.docx]

**Table S2.** List of primers used for pathogen specific PCR

| Target pathogen | Name | Sequences (5' - 3') | Cycling condition |
| --- | --- | --- | --- |
| B19 | e1905f | TGCAGATGCCCTCCACCCA | 45 cycles of 95^o^C for 30 seconds, 60^o^C for 1 minute. |
|  | e1987r | GCTGCTTTCACTGAGTTCTTC |  |
| HBV | SP1s | GCTCCGACTATTGCCTCTCTCACA | 45 cycles of 95^o^C for 30 seconds, 60^o^C for 1 minute. |
|  | SP1a | TGTAACACGAGAAGGGGTCCTAGGA |  |
| DENV | D1 | TCAATATGCTGAAACGCGCGAGAAACCG | 1st PCR (primer D1 and D2): 40 cycles of 94^o^C for 30 seconds, 55^o^C for 1 minute, 72^o^C for 2 minutes. 2nd PCR (primer D1 and TS1/TS2): 20-25 cycles f 94^o^C for 30 seconds, 55^o^C for 1 minute, 72^o^C for 2 minutes. |
|  | D2 | TTGCACCAACAGTCAATGTCTTCAGGTTC |  |
|  | TS1 | CGTCTCAGTGATCCGGGGG |  |
|  | TS2 | CGCCACAAGGGCCATGAACAG |  |
| HIV-1 | 6F-HIV | CATGTTTTCAGCATTATCAGAAGGA | 45 cycles of 95^o^C for 30 seconds, 60^o^C for 1 minute. |
|  | 84R-HIV | TGCTTGATGTCCCCCCACT |  |
|  | HIV-intF | CCCTACAATCCCCAAAGTCA | 35 cycles of 95^o^C for 30 seconds, 55^o^C for 30, 72^o^C for 30 seconds. |
|  | HIV-intR | CTTGCCACACAATCATCACC |  |
|  | SK39 | TTTGGTCCTTGTCTTATGTCCAGAATGC | 1st PCR (primer SK39 and SK145): 45 cycles of 95^o^C for 30 seconds, 60^o^C for 1 minute. 2nd PCR (primer SK39 and SK101): 25 cycles of 95^o^C for 30 seconds, 60^o^C for 1 minute. |
|  | SK101 | GCTATGTCAGTTCCCCTTGGTTCTC |  |
|  | SK145 | AGTGGGGGGACATCAAGCAGCCATGCAAAT |  |
|  | HIV1-outer-F | AGGGCTGTTGGAAATGTGGA | 1st PCR (outer primer): 45 cycles of 95^o^C for 30 seconds, 60^o^C for 1 minute. 2nd PCR (inner primer): 25 cycles of 95^o^C for 30 seconds, 60^o^C for 1 minute. |
|  | HIV1-outer-R | ACGTTGACAGGTGTAGGTCC |  |
|  | HIV1-inner-F | TGGAAATGTGGAAAGGAAGG |  |
|  | HIV1-inner-R | GCCAAAGAGTGATTTGAGGGC |  |

B19, human parvovirus B19; HBV, hepatitis B virus; DENV, dengue virus; HIV, human immunodeficiency virus.
