# Supplementary material for: Circular Whole-Transcriptome Amplification (cWTA) and mNGS Screening Enhanced by a Group Testing Algorithm (mEGA) Enable High-Throughput and Comprehensive Virus Identification
Source: mSphere. 2022 Aug 25;7(5):e00332-22. doi: 10.1128/msphere.00332-22 (PMC9599668; doi:10.1128/msphere.00332-22)
Supplement: TABLE S4 [file msphere.00332-22-s0008.docx]

**Table S4.** Sequencing reads from the pooled experiment aligned to parvovirus B19 and the subsequent threshold calculation

| Pool | B19 reads |
| --- | --- |
|  |  |
| 1a | **4** |
| 1b | 47,820 |
| 2a | 34,861 |
| 2b | 0 |
| 3a | **5** |
| 3b | 73,521 |
| 4a | **31** |
| 4b | 46,792 |
| 5a | 96,198 |
| 5b | 0 |
| 5c | 0 |
| Total viral read | 299,232 |
| Index hopping rate (%) | 0.1 |
| λ* | 299.232 |
| positive threshold (≥) | 341 |

Bold numbers indicate number below the threshold. *The λ value was calculated with assumption that index hopping rate is equal to 0.1% (28).
